# Oxygen Regulates Human Pluripotent Stem Cell Metabolic Flux

**DOI:** 10.1155/2019/8195614

**Published:** 2019-05-19

**Authors:** Jarmon G. Lees, Timothy S. Cliff, Amanda Gammilonghi, James G. Ryall, Stephen Dalton, David K. Gardner, Alexandra J. Harvey

**Affiliations:** ^1^School of BioSciences, The University of Melbourne, 11 Royal Parade, Parkville, 3010 VIC, Australia; ^2^Department of Biochemistry and Molecular Biology, University of Georgia, 500 D.W. Brooks Drive, Athens, GA 30602, USA; ^3^Centre for Molecular Medicine, University of Georgia, 500 D.W. Brooks Drive, Athens, GA 30602, USA; ^4^Nanobiotechnology Research Laboratory, RMIT University, Melbourne, VIC 3010, Australia; ^5^Centre for Muscle Research, Department of Physiology, The University of Melbourne, Melbourne, VIC 3010, Australia

## Abstract

Metabolism has been shown to alter cell fate in human pluripotent stem cells (hPSC). However, current understanding is almost exclusively based on work performed at 20% oxygen (air), with very few studies reporting on hPSC at physiological oxygen (5%). In this study, we integrated metabolic, transcriptomic, and epigenetic data to elucidate the impact of oxygen on hPSC. Using ^13^C-glucose labeling, we show that 5% oxygen increased the intracellular levels of glycolytic intermediates, glycogen, and the antioxidant response in hPSC. In contrast, 20% oxygen increased metabolite flux through the TCA cycle, activity of mitochondria, and ATP production. Acetylation of H3K9 and H3K27 was elevated at 5% oxygen while H3K27 trimethylation was decreased, conforming to a more open chromatin structure. RNA-seq analysis of 5% oxygen hPSC also indicated increases in glycolysis, lysine demethylases, and glucose-derived carbon metabolism, while increased methyltransferase and cell cycle activity was indicated at 20% oxygen. Our findings show that oxygen drives metabolite flux and specifies carbon fate in hPSC and, although the mechanism remains to be elucidated, oxygen was shown to alter methyltransferase and demethylase activity and the global epigenetic landscape.

## 1. Introduction

Oxygen is a critical, but often overlooked, metabolite within the stem cell niche. The mammalian reproductive tract, within which the preimplantation embryo develops, has been measured at 2-9% oxygen in the rat, rabbit, hamster, and rhesus monkey [1, 2]. The precise oxygen concentration experienced by the inner cell mass of the human blastocyst is unknown but likely approximates less than 5% [3–5]. In contrast, atmospheric (20%) oxygen remains the predominant concentration used for cell culture, including stem cells and human embryos, despite documented evidence of its detrimental impact [6], with limited adoption of more physiological oxygen concentrations [7].

PSC metabolism is characterized by a heavy dependenceon glycolysis, with ~50-70% of glucose being converted to lactate [8–11]. In contrast, mitochondrial oxidative phosphorylation (OXPHOS) occurs at relatively low levels in PSC compared to their differentiated counterparts [9, 12]. When cultured at 5% oxygen, hPSC increase the flux of glucose through glycolysis [11, 13, 14], increase glycolytic gene expression [11, 15], and decrease oxidative gene expression [14]. Oxygen has also been shown to regulate hPSC mitochondrial activity and biogenesis [14], as it occurs in somatic cells [16]. Physiological oxygen conditions therefore establish a PSC metabolic state characterized by increased glucose/lactate flux and suppressed mitochondrial biogenesis and activity relative to PSC developed in atmospheric oxygen. Glycolytic and mitochondrial flux in PSC also generates key epigenetic cofactors acetyl-CoA, alpha-ketoglutarate (*α*KG), NADH/NAD^+^, and TCA intermediates including citrate and succinate, which maintain the highly acetylated euchromatic landscape characteristic of pluripotency, while in contrast, differentiated cell types are generally heterochromatic [17]. Currently, only one study has looked at the impact of oxygen on hPSC epigenetic markers [18], in which 5% oxygen induced a euchromatic state in the chromatic configuration around the hypoxic response elements (HREs) of *OCT4*, *SOX2*, and *NANOG* pluripotency transcriptional promotors, leading to increased levels of H3K36me3, decreased H3K9me3, and a general increase in pluripotency markers, however none have integrated metabolic, epigenetic and transcriptomic data within a single culture system.

Oxygen is a key factor, not just in PSC culture, but in all cell, tissue, and organ cultures. However, our understanding of how oxygen acts as a nutrient and signaling molecule in PSC is limited. Because metabolism is inextricably linked to epigenetics [19–21] and, therefore, cell fate [17], it is important to define the role of oxygen in metabolic pathways. By using extracellular acidification rates (ECAR) and oxygen consumption rates (OCR) as measures of glycolysis and OXPHOS, respectively, we find that physiological (5%) oxygen levels decrease mitochondrial activity and increase glycolytic flux in hPSC. This was confirmed with heteronuclear single-quantum coherence (HSQC) nuclear magnetic resonance (NMR) spectroscopy of hPSC labeled with ^13^C-glucose. Oxygen is therefore a key regulator of hPSC metabolism, directing the flux of glucose-derived carbon to lactate, alanine, glycogen, and glutathione at physiological oxygen and through the tricarboxylic acid (TCA) cycle to acetate at 20% oxygen. Coincident with altered intracellular metabolite flux, 5% oxygen increased acetylation and decreased methylation markers consistent with a more active gene regulatory network. RNA-seq analysis confirmed increases in transcription and glycolytic metabolism and confirmed the resulting epigenetic landscape at physiological oxygen levels. These findings show that 5% oxygen has a significant impact on intracellular metabolite fluxes in hPSC, metabolites which are known epigenetic cofactors, accompanied by increased H3K9/27ac and decreased H3K27me3. Although the mechanism remains to be elucidated, oxygen was shown to alter methyltransferase and demethylase activity influencing the global epigenetic landscape.

## 2. Results

### 2.1. Mitochondrial Activity Is Decreased at Physiological Oxygen

Mitochondrial metabolism is often considered negligible in hPSC [10, 22–24], as they are known to rely heavily on the glycolytic oxidation of glucose to lactate in the cytosol to meet their energy and biosynthetic needs [9, 19], and pluripotency is reportedly increased when the mitochondria are inhibited with antimycin A [22]. Paradoxically, attenuation of specific mitochondrial functions leads to apoptosis, tumourigenesis, or the loss of pluripotency. [25]. Significantly, the role of the hPSC mitochondria, the largest consumers of oxygen in the cell, has never been addressed under physiological oxygen conditions. Indeed, our current understanding of mitochondria, metabolism, and pluripotency is primarily based on work performed at 20% oxygen. To address this, we assessed the metabolic pathway activity of hPSC cultured under standard (20%) and physiological (5%) oxygen conditions using oxygen consumption and extracellular acidification rates.

**Figure 1:**
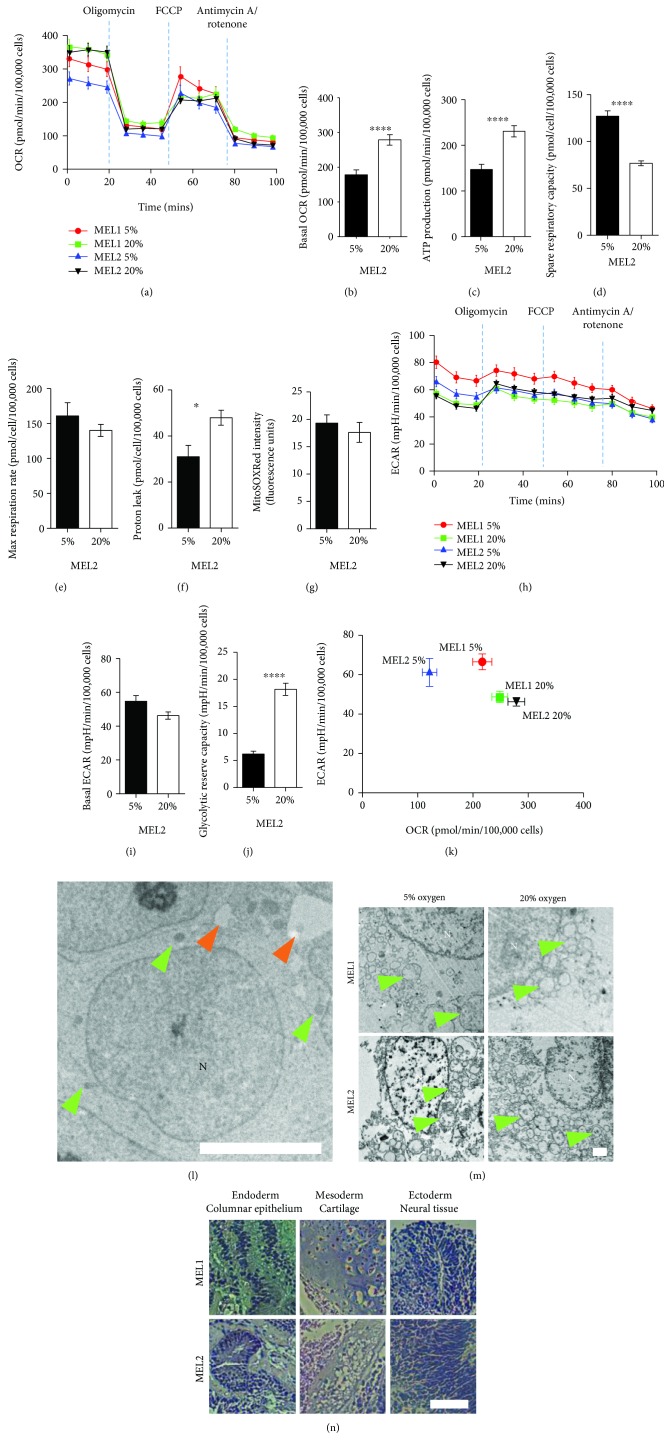
Physiological oxygen promotes a glycolytic metabolism in hPSC. (a) Oxygen consumption rate (OCR) analysis of MEL1 and MEL2 hPSC cultured under 5% and 20% oxygen conditions, assessed using oligomycin (1 *μ*M), FCCP (0.3 *μ*M), and antimycin A/rotenone (1 *μ*M). (b–f) Basal OCR (b), ATP production (c), spare respiratory capacity (d), max respiration rate (e), and proton leak (f) as determined from OCR data. (g) Mitochondrial superoxide levels in MEL2 hPSC. (h) Extracellular acidification rate (ECAR) analysis of MEL1 and MEL2 hPSC cultured at 5% and 20% oxygen. (i–j) Basal ECAR (i) and glycolytic reserve capacity (j) determined from ECAR data. (k) Metabolic phenogram contrasting basal OCR and ECAR. (l) Transmission electron micrographs (TEM) of hPSC showing the nuclei (N), mitochondria (green arrows), and lipid droplets (orange arrows). (m) High magnification TEM of MEL1 and MEL2 hPSC identifying the mitochondria (green arrows) and their inner mitochondrial matrix development. (n) hPSC-derived in vitro teratomas showing ectoderm, mesoderm, and endoderm lineages. All assays performed in biological triplicate. Error bars represent the SEM. ^∗∗∗∗^ *p* < 0.0001, ^∗∗∗^ *p* < 0.001, ^∗∗^ *p* < 0.01, and ^∗^ *p* < 0.05 for 2-factor ANOVA flowed by simple main effects analyses. Scale bars: 5 *μ*m (l), 2 *μ*m (m), and 100 *μ*m (n).

Compared with hPSC cultured at 20% oxygen, those cultured at 5% had lower OCR signifying a lower resting mitochondrial activity (Figures 1(a) and 1(b)). Furthermore, cells cultured at 5% oxygen had reduced rates of oligomycin-sensitive oxygen consumption, consistent with a lower dependence on OXPHOS, and a lower rate of ATP production (Figure 1(c)). hPSC cultured at 5% oxygen were found to actively suppress mitochondrial activity (Figure 1(d)) but were able to match the maximum OCR of those grown at 20% oxygen when treated with the mitochondrial uncoupler FCCP (Figure 1(e)). hPSC at 20% oxygen moderated a higher basal OCR by increasing mitochondrial H^+^ leak (Figure 1(f)), a strategy which limits not only ATP production but also ROS production [26]. Indeed, ROS production was not different under either oxygen condition (Figure 1(g)). Surprisingly, resting ECAR was not affected by 5% and 20% oxygen (Figure 1(i)); however, hPSC at 20% had almost four times the glycolytic reserve capacity of cells cultured at 5% (Figure 1(j)) indicating an active suppression of lactate flux at 20% oxygen. Taken together, the metabolic phenotype of hPSC grown at 5% oxygen is more glycolytic with active suppression of mitochondrial oxidative metabolism, while the metabolic phenotype at 20% oxygen makes more use of mitochondrial oxidation and has an active suppression of glycolytic metabolism (Figure 1(k)).

Clear differences in the metabolic profiles of hPSC due to oxygen in culture prompted the analysis of mitochondrial morphology by transmission electron microscopy (TEM), which has been related to mitochondrial function [9]. Irrespective of oxygen concentration, near-spherical mitochondria clustered perinuclearly and towards one pole of the nucleus with almost no evidence of elongation or reticulation (Figure 1(l)). Within the cytoplasm, the mitochondria were the most prominent organelles. At higher magnification, the hPSC mitochondrial crista structure was chaotic and disconnected lacking inner-membrane-to-outer-membrane organization, again with no apparent effect of oxygen (Figure 1(m)). This morphology is not surprising, given that the dominant mitochondrial morphology of the embryo's inner cell mass (ICM) cells is spherical, with clear matrices and few peripheral arched cristae [21, 27]. To characterize the pluripotency of the hPSC lines, we used an in vitro teratoma assay [28] to determine the germ layer differentiation potential. After 8 weeks in the presence of serum, both lines differentiated into teratomas with evidence of endoderm, mesoderm, and ectoderm tissues (Figure 1(n)). Standard pluripotency transcripts have been assessed previously and were not different due to oxygen [14]. Using ECAR, OCR, mitochondrial ROS levels, and mitochondrial morphology, we show that 5% oxygen decreases mitochondrial activity and increases flux from glucose to lactate in hPSC. These observations raised questions about the fate of glucose-derived carbon in hPSC cultured at physiological and nonphysiological oxygen.

### 2.2. Intracellular Metabolite Accumulation Is Regulated by Oxygen

**Figure 2:**
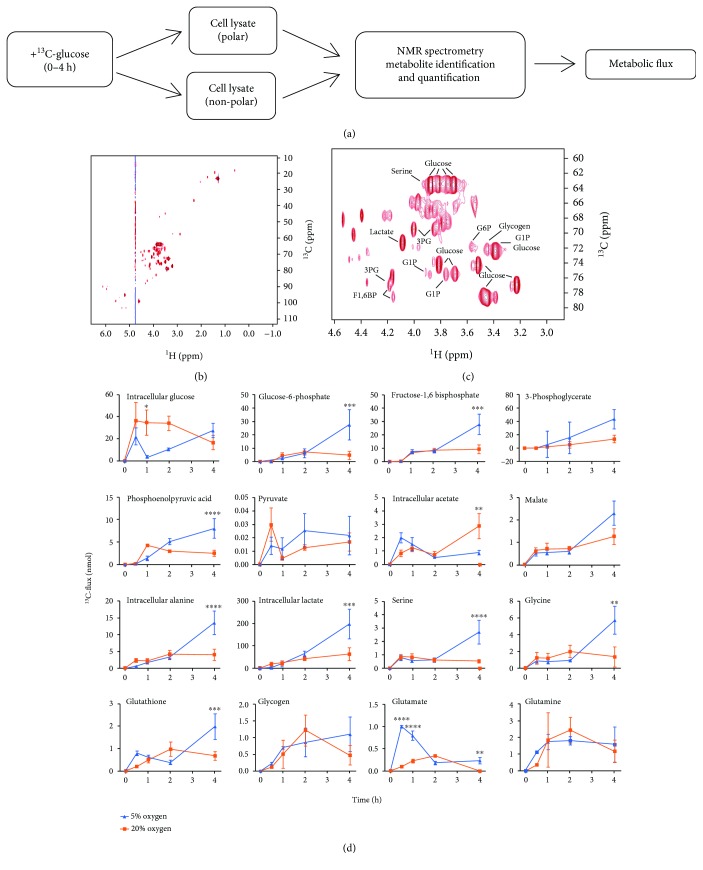
Intracellular glucose-derived metabolites accumulate more rapidly under physiological oxygen. (a) ^13^C-glucose fluxomics workflow using NMR. (b–c) Representative 2D ^1^H-^13^C HSQC NMR spectra (b). Magnification of NMR spectrum showing representative metabolites (c). Each peak is a quantifiable carbon-hydrogen bond from a metabolite. (d) ^13^C-flux analysis over 0–4 hours of MEL2 hPSC cultured under 5% or 20% oxygen. Measurements are of intracellular metabolite levels. All assays performed in biological triplicate. Error bars represent the SEM. ^∗∗∗∗^ *p* < 0.0001, ^∗∗∗^ *p* < 0.001, ^∗∗^ *p* < 0.01, and ^∗^ *p* < 0.05 for paired Student's *t*-test.

Physiological (5%) and atmospheric (20%) oxygen results in distinct global metabolic profiles characterized by increased glycolytic and mitochondrial metabolism, respectively. To determine the underlying metabolite fluxes that contribute to these phenotypes, we performed metabolic labelling with uniformly labeled ^13^C-glucose, followed by NMR-based fluxome analysis [29]. This methodology delivers a quantitative and temporal description of the metabolic landscape. Because we have demonstrated here, and previously by extracellular metabolite profiling [11, 14, 30], that oxygen impacts glycolytic flux, we focused primarily on this pathway for ^13^C flux analysis. hPSC adapted to either 5% or 20% oxygen culture were labeled with ^13^C-glucose over 0-4 h, and H,C-HSQC NMR spectroscopy was performed on polar and nonpolar cell fractions (Figure 2(a)). This time course allows us to track the fate of ^13^C, the source of metabolites contributing to glycolytic fluxomes and pathway utilization, without the saturation problems associated with longer periods of metabolic labeling [19, 29], or the limitations of endpoint metabolic analyses as performed previously.. Representative spectra are shown in Figures 2(b) and 2(c). Following labeling with ^13^C-glucose, we quantified the levels of ^13^C-labeled metabolites (Figure 2(d)). The analysis included the quantitation of metabolites in the glycolytic pathway, tricarboxylic acid (TCA) cycle, transhydrogenase cycle, glutamate-glutamine cycle, and generation of glutathione (GSH), acetate, glycogen, and amino acids. For example, we have previously measured the extracellular levels of alanine and lactate which were unchanged and increased, respectively, in response to 5% oxygen [14]. In the current study, we observed that intracellular accumulations of both alanine and lactate were unchanged by oxygen between 0 and 2 h but both increased 3-fold between 2 and 4 h (Figure 2(d)). Temporal fluxomics, therefore, is a powerful tool for identifying underlying metabolite fluxes prior to nutrient depletion over time. This analysis can also highlight key points of differential metabolic regulation due to oxygen. For example, our analysis suggests that hexokinase (HK) activity is saturated at 20% oxygen, indicated by the accumulation of intracellular glucose relative to glucose-6-phosphate (Figure 2(d)). This pattern is not observed at 5% oxygen, strongly suggesting that HK activity is oxygen regulated. This analysis shows that rates of metabolite accumulation associated with glycolysis are higher at 5% oxygen. Intracellular lactate and alanine showed a similar pattern (Figure 2(d)). Acetate and malate, TCA cycle metabolites, were also quantified (Figure 2(d)) where the only metabolite that accumulated more at 20% oxygen was acetate, indicating that flux through the TCA cycle may be elevated at 20% oxygen relative to 5% oxygen in hPSC. These data are consistent with ECAR and OCR analyses in Figure 1 showing elevated glycolysis at 5% oxygen and elevated mitochondrial oxidation at 20% oxygen.

### 2.3. Metabolic Flux Is Greater at Physiological Oxygen

**Figure 3:**
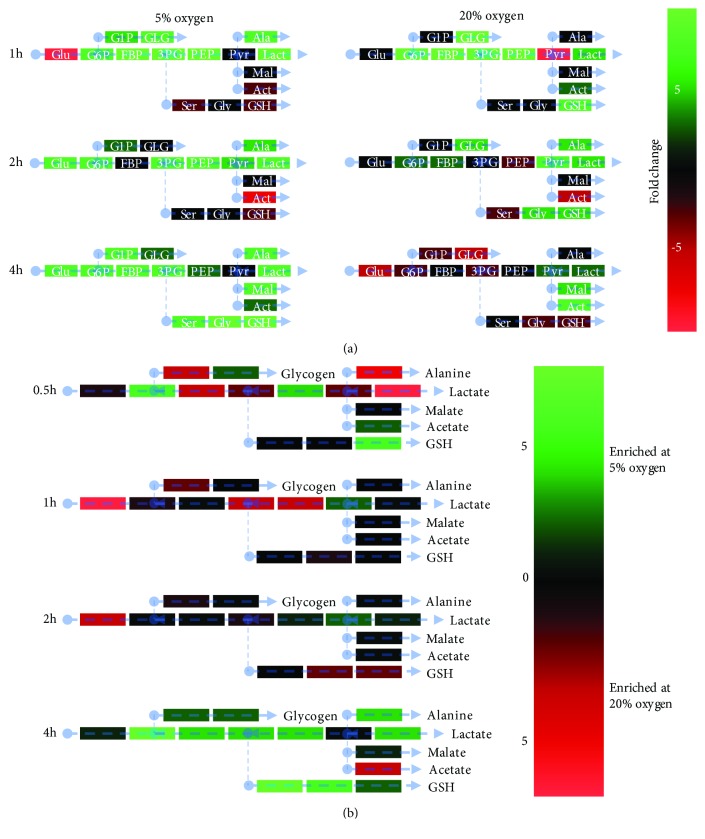
5% oxygen stimulates glycolytic, glutathione, glycogen, and alanine accumulation in hPSC over time. (a) Heatmap comparing intracellular metabolite levels to those of the previous timepoint, organized into functional groups. Data are the fold change. Green indicates an increase from the previous timepoint. Red indicates a decrease from the previous timepoint. (b) Heatmap showing the enrichment of intracellular metabolites between hPSC cultured at 5% and 20% oxygen at each timepoint. Green indicates an enrichment at 5% relative to 20% oxygen. Red indicates an enrichment at 20% relative to 5% oxygen. All assays performed in biological triplicate.

Heatmaps of metabolite accumulation over time within oxygen treatments describe a rapid increase in all metabolic pathways at 5% oxygen, primarily between 2 and 4 h (Figure 3(a)), indicating hPSC have a faster metabolic rate under physiological oxygen conditions. At 20% oxygen, however, overall metabolite accumulation slowed, with only lactate and the TCA cycle metabolites malate and acetate increased between 2 and 4 h (Figure 3(a)) suggesting diversion of glucose products to alternative pathways. Contrasting oxygen conditions directly at each timepoint (Figure 3(b) and Supplementary Figure S1, glucose-derived intracellular metabolite levels were not significantly different between 0 and 2 h; however, at 5% oxygen, increased lactate, alanine, glycogen synthesis, and GSH production were observed by 4 h. These data clearly show that intracellular metabolites are oxygen regulated, plausibly by oxygen-sensitive metabolic enzymes. ROS production was not increased by low oxygen as has been suggested [21, 31], although levels of GSH and its precursors serine and glycine were higher, plausibly mitigating ROS levels. These findings confirm that oxygen, an easily controllable, physiologically relevant nutrient in cell culture, plays a leading role in defining the intracellular metabolite landscape in hPSC.

### 2.4. Physiological Oxygen Promotes H3K9/H3K27 Acetylation and Represses H3K27 Trimethylation

**Figure 4:**
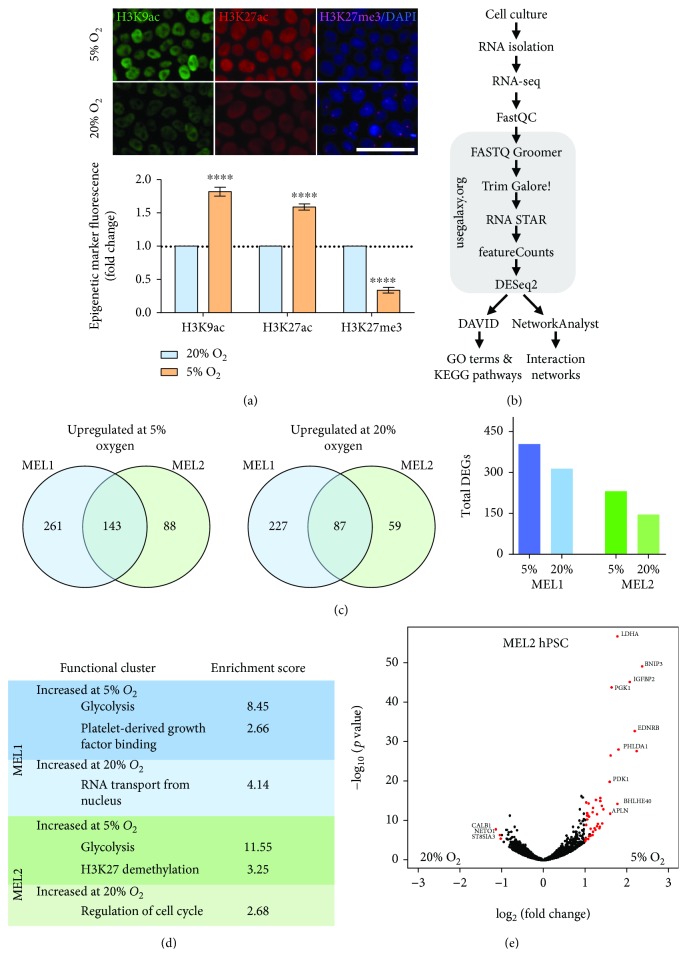
Global acetylation is increased, and methylation is decreased at 5% oxygen. (a) H3K9ac, H3K27ac, and K3K27me3 fold change of MEL2 hPSC at 5% relative to 20% oxygen. Scale is 50 *μ*m. (b) Workflow for RNA-seq analysis using https://usegalaxy.org/, DAVID, and NetworkAnalayst. (c) Venn diagrams showing the overlap of genes upregulated in two hPSC lines at 5% oxygen and 20% oxygen and total DEG count. (d) Functional clusters with enrichment scores greater than 2 derived from *Biological Process* and *Molecular Function* GO terms. (e) Volcano plot of MEL2 hPSC transcriptional response to 5% and 20% oxygen. Red genes indicate a fold change value greater than 2 and an adjusted *p* value (Benjamini FDR) less than 0.05. All assays performed in biological triplicate. Error bars represent the SEM. ^∗∗∗∗^ *p* < 0.0001 for paired Student's *t*-test.

We have shown that oxygen controls the direction and magnitude of glucose-derived carbon flux within hPSC. Several metabolites which can be derived from glucose are epigenetic modifiers and cofactors which are required for the modulation of the epigenetic landscape [17, 32]. Global acetylation levels of H3K9 and H3K27 increased ~75% in hPSC cultured at 5% oxygen relative to those at 20% oxygen (Figure 4(a)). Global trimethylation of the transcriptional repressor H3K27 was 3-fold lower at 5% oxygen (Figure 4(a)). Taken together, this epigenetic profile is consistent with a more open chromatin structure at physiological oxygen [18]. RNA-seq of hPSC cultured long-term at 5% and 20% oxygen (Figure 4(b) and Supplementary Table 1) confirmed a more active transcriptome at 5% oxygen. Upregulated differentially expressed gene (DEG) counts were higher at 5% oxygen in the two hPSC lines assessed (Figure 4(c)). The cluster analysis of DEGs was consistent with ^13^C flux analyses (Figure 4(d)), highlighting glycolysis as the most highly enriched functional cluster at 5% oxygen, followed by H3K27 demethylation. Glycolytic genes upregulated at 5% oxygen included *LDHA*, *PGK1*, and glucose transporters *SLC2A1* (*GLUT1*) and *SLC2A14* (*GLUT14*) (Figure 4(e)). Several of these transcripts have been independently validated by qPCR and microarray and confirmed to be oxygen-regulated in a consistent manner with the current findings [11, 15]. The RNA-seq data also confirms the increased carbon flux through glycolytic pathways (Figure 2(d)) and reduced H3K27me3 staining (Figure 4(a)) observed at 5% oxygen.

Further interrogation of RNA-seq data revealed the overall transcriptional response to oxygen conformed to a more open chromatin structure at 5% oxygen (Figures 4(e) and S2). In response to 5% oxygen, 46 and 47 DEGs were upregulated >2-fold in MEL2 and MEL1 hPSC lines, respectively. Transcripts upregulated at 5% oxygen in addition to the glycolytic genes mentioned previously include proteins that contribute to extracellular matrix organization (*PCDH10*, *FBN1*, *VCAN*, and *POSTN*), pro-apoptotic factors in response to mitochondrial damage (*BNIP3*), cell cycle (*BTG2*), early embryonic methylation patterning (*BORIS*), and signal transducers including hypoxia inducible factor- (HIF-) related genes (*ANXA3*, *GDF15*, *IFGBP2*, and *IFGBP5*). In response to culture at 20% oxygen, only 3 and 2 DEGs were upregulated >2-fold in the MEL2 and MEL1 hPSC lines, respectively. Of interest, follistatin (*FST*) which has a high affinity for activin A and leads to hPSC differentiation [33] was increased at 20% oxygen (Supplementary Figure S2). Consistent with previous reports [11, 14, 34–36], the remodeling of the metabolome did not coincide with a pronounced change in the majority of pluripotency markers. Pluripotency markers *OCT4*, *NANOG*, *MEIS1 OTX2*, *SOX11*, *GDF3*, *REX1*, *FGF4*, *DPPA2*, *DPPA4*, and *hTERT* were unaltered due to oxygen (Supplementary Figure S3A). At 5% oxygen, the expression of the pluripotency marker *OCT6* (*POU3F1*) [37] was increased in both lines, while *SOX2* and *DNMT3B* were decreased in the MEL1 and both hPSC lines, respectively (Supplementary Figure S3). However, none of these genes reached the log_2_(fc)=1 threshold.

**Figure 5:**
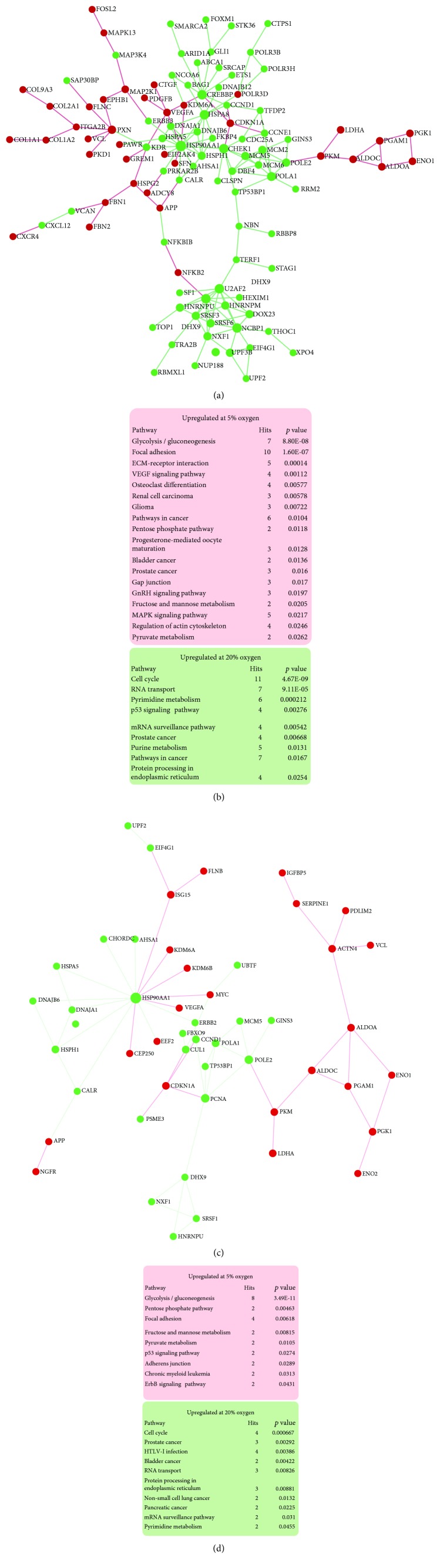
RNA-seq zero-order networks in hPSC due to 5% and 20% oxygen culture. (a) MEL1 hPSC differentially expressed genes due to oxygen were submitted to NetworkAnalyst and connected based on known protein:protein interactions. Red nodes are upregulated at 5% oxygen; green nodes are upregulated at 20% oxygen. Green/red intensity indicates the degree of fold change as indicated in the figure. (b) MEL1 hPSC KEGG pathways upregulated at 5% and 20% oxygen based on the zero-order network in Figure 5(a). (c) MEL2 hPSC zero-order and protein:protein interaction network. (d) MEL2 hPSC KEGG pathways upregulated at 5% and 20% oxygen based on the zero-order network in (c).

In order to understand more the transcriptional response to oxygen, we interrogated RNA-seq data to identify oxygen-regulated networks based on the identified DEGs. NetworkAnalyst [38] was used to generate zero-order protein-protein networks based on known protein interactions of significant DEGs (Figures 5(a)–5(d)). Subsequent KEGG pathway analysis of hPSC networks upregulated at 5% oxygen highlighted glycolysis most prominently, followed by focal adhesion and pentose phosphate pathway activity (Figures 5(b) and 5(d)). In hPSC cultured at 20% oxygen, cell cycle was the most enriched pathway, followed by RNA transport and protein processing in the endoplasmic reticulum. These pathway enrichments based on known networked interactions were similar to GO and KEGG analyses performed using all DEGs, in which glycolysis, amino acid and carbon metabolism, and the HIF-1 hypoxic response were enriched at 5% oxygen (Supplementary Figures S3B and S4). As expected, *HIF-1α*, *HIF-1β*, and *HIF-2α* were not oxygen regulated at the mRNA level; however, HIF-1 target genes *ENO1*, *SERPINE1*, *ENO2*, *VEGFA*, *SLC2A1*, *EGLN1*, *PDK1*, *HK2*, *MAP2K1*, and *PFKFB3* were significantly increased at low oxygen (Supplementary Figure S3B). At 20% oxygen, terms related to cell cycle transitions were consistently enriched despite no evidence that oxygen affects the proliferative capacity of either of the hPSC lines used [14]. Under both oxygen conditions, chemokine signaling was decreased in the MEL1 line relative to that in the MEL2 line (Supplementary Figures S5A–S5D).

Together, these results demonstrate that a physiological oxygen concentration of 5% is able to globally increase acetylation and decrease methylation levels, plausibly responsible for the significant transcriptional response observed and consistent with a more open chromatin structure. This response not only includes the expected glycolytic and hypoxic response transcripts but is also enriched for genes in a range of metabolic pathways and those that contribute to the extracellular matrix. In contrast, an atmospheric oxygen concentration of 20% results in a repressed hPSC epigenetic profile as indicated by lower acetylation, higher methylation, and lower transcriptional activity. Overall, these results support a role for oxygen in determining the hPSC epigenetic landscape and implicate metabolites in this process, downstream of the change in oxygen.

### 2.5. Concurrent Methyltransferase and Demethylase Regulation by Oxygen

The results presented in the preceding paragraphs indicate that a change in oxygen is sufficient to alter hPSC histone methylation status. Next, we examined the transcript levels of methyl donors through the serine/glycine biosynthesis pathway and downstream methionine and folate cycles using RNA-seq data and qPCR (Supplementary Figure S6 and Supplementary Table 1). Glycolytic enzymes *LDHA*, which converts pyruvate to lactate, and *PGK1*, which catalyzes 1,3-bisphosphoglycerate metabolism to 3-phosphoglycerate (3PG), were also increased (Supplementary Figure S6A and Supplementary Table 1). 3PG marks the crossroads where carbon can either continue through glycolysis or be directed into the serine/glycine pathway. While intracellular levels of serine and glycine were increased at 5% oxygen, enzymes necessary for their synthesis were not regulated by oxygen (Supplementary Figure S6B). Plausibly, the mass effect of carbon flux through glycolysis is sufficient to increase serine/glycine production. Subsequent flux through the transsulphuration pathway to form cysteine and GSH is increased despite lower levels of *CBS* at 5% oxygen (Supplementary Figure S6C). Quantification by qPCR established that approximately half of the assessed enzyme transcript levels in the folate and methionine cycles were decreased at 5% oxygen, while the remaining genes were unaltered (Supplementary Figures S6D and S5E and Supplementary Table 1). From the RNA-seq analysis, folate cycle enzyme transcripts for *MTHFR* and *MTHFD1L* were downregulated at 5% oxygen, while *SHMT2* and *GLDC* were unaltered (Supplementary Figure S6D). In the methionine cycle, transcripts for *MAT2A*, *DNMT1*, and *DNMT3B* were downregulated, while those for *MAT2B*, *DNMT3A*, and *DNMT3L* remained unchanged (Supplementary Figure S6E). Consistent with a reduction in histone methylation, *MAT2A* was decreased at 5% oxygen. MAT2A interacts with the histone-lysine *N*-methyltransferase EZH2 [39], which also decreased at 5% oxygen (Supplementary Figure S6F), to specifically methylate H3K27 [40]. MAT2A is also responsible for the formation of the methyl donor S-adenosylmethionine (SAM) from methionine [32, 41], which donates a methyl group to histones or DNA. As*DNMT1* and *DNMT3B* were likewise downregulated at 5% oxygen, they may in part be responsible for reducing DNA methylation capacity although this was not assessed. Finally, in parallel with reduced histone methylation capacity through reduced *EZH2*, *MAT2A* expression and reduced global H3K27me3 (Figure 4(a)), RNA-seq analysis showed that lysine demethylases (KDMs) which remove methyl groups from histones were upregulated at 5% oxygen (Supplementary Figure S6F). *Lysine demethylase 7A* (*KDM7A*), *KDM6A*, *KDM4B*, and *KDM3A* were upregulated by more than 50%, while *KDM6B*, which specifically demethylates H3K27me3 [42, 43], was upregulated by 260% (Supplementary Table 1). Overall, these findings begin to show a link between a change in oxygen and the hPSC epigenetic landscape, suggesting a role for oxygen-regulated metabolites and enzymes in the process.

## 3. Discussion

Previous analyses of hPSC metabolism and its role in pluripotency have been described in cells cultured under 20% oxygen (5% CO2 in air; atmospheric). We have established that hPSC mitochondrial respiration is decreased at 5% oxygen, despite the absence of any apparent alterations to inner mitochondrial matrix organization. Beyond the production of ATP, mitochondrial respiration also generates ROS as H_2_O_2_ from complex III of the electron transport chain [44], which are thought to increase under conditions of low oxygen [31]. Compared with their better known role in DNA damage when in excess, ROS have been shown to directly alter the epigenetic landscape [45] by directly oxidising the methyl group of 5mC preventing methylation [46] and inducing DNA methylation at the promotor regions of acetyltransferases and methyltransferases [47]. Our study, however, reveals that a physiological concentration of oxygen did not elicit a change in steady-state ROS levels. Instead, intracellular glutathione production was doubled, plausibly mitigating any excess ROS production. The oxygen-regulated mechanisms driving hPSC acetylation and methylation may therefore be related to the altered metabolite fluxes we observed in this study.

A euchromatic state was established at 5% oxygen, consisting of increased global H3K9/27 acetylation and reduced H3K27 trimethylation. Metabolite levels and pathway fluxes alter the epigenetic landscape, potentially regulating global or localized patterns of genes to elicit cell fate [20, 21, 48, 49]. We show that 5% oxygen increases glucose-derived carbon flux, which feeds into serine/glycine biosynthesis, glycogen, lactate, alanine, and glutathione production. Despite elevated levels of serine, glycine, and glutathione at 5% oxygen, we observed depressed expression of enzymes throughout the folate and methionine cycles, which are necessary for the production of key methyltransferases such as SAM and EZH2 [40, 41]. *MAT2A*, but not *MAT2B* expression, which together generate EZH2 and SAM from methionine, was also downregulated at 5% oxygen. In support of this, an identical *MAT2A/B* expression pattern is observed when methionine is removed from hPSC culture [32], leading to a reduction in SAM synthesis and a reduction in global methylation consistent with our findings. The repressed expression of key methyltransferase enzymes and the corresponding decrease in methylation at 5% oxygen is consistent with the profile of enhanced pluripotency [17]. Indeed, the acquisition of naïvety in mouse PSC is associated with demethylation events, specifically of H3K27me3 [19]. While oxygen did not generally affect pluripotency at a transcriptional level, consistent with several previous assessments [11, 14, 34–36], physiological oxygen was shown to promote a metabolome and epigenetic landscape characteristic of pluripotency. It is plausible that HIF signaling is partly responsible for the metabolic profile and therefore shift in physiology established at 5% oxygen, as over half of the upregulated HIF target genes function in glycolysis, consistent with the observed increases in glycolytic metabolism.

A question arising from our work is why 5% oxygen resulted in the lower accumulation of intracellular acetate coincident with higher global H3K9/27 acetylation. Of all assessed metabolites, only acetate, an essential metabolite for most cancers [50], was less present under 5% oxygen culture. Upon hPSC differentiation, global H3K9/27ac is lost; however, acetate supplementation is able to completely reverse this [17]. Notably, acetylation by acetate is achieved only after its conversion to acetyl-CoA, which is primarily formed through glycolytic-derived pyruvate. Pyruvate is metabolised to citrate in the mitochondria which is then exported to the cytosol where it is metabolised to acetyl-CoA [51], thus bypassing acetate. The lower accumulation of glucose-derived acetate at 5% oxygen may therefore signal a higher demand for acetyl-CoA for acetylation purposes.

Several key epigenetic cofactors can be derived from glucose metabolism and, thus, are potentially influenced by oxygen. The production of cytosolic acetyl-CoA requires glycolytic metabolism of citrate, while acetyl-CoA produced in the mitochondria from fatty acids is metabolised through the TCA cycle [51]. The current study cannot definitively conclude that the increased acetylation is glucose-derived; however, increased carbon flux through glycolysis such as that observed in this study at 5% oxygen has been shown to reduce deacetylase activity through reduced NAD^+^ availability [52] and increase acetylation through glucose-derived acetyl-CoA [17]. Conversely, the observed reduction in histone methylation at 5% oxygen is likely due to the global increase in histone demethylases and general decrease in methyltransferase enzyme transcripts *MAT2A* and *EZH2* [53]. Furthermore, the downregulation of DNMTs at 5% oxygen is consistent with the global DNA methylation pattern seen in the 4-cell and blastocyst stage bovine embryo in response to oxygen [54], suggesting that the totipotent and pluripotent developmental stages represent heightened windows of sensitivity to oxygen concentration. Taken together, these data implicate the higher glycolytic flux generated at 5% oxygen in both the accumulation of acetylation and loss of methylation marks. These findings could be enhanced by tracing the origin of the acetyl groups on histone tails.

In this report, we show that physiological oxygen (5%) promotes a euchromatic state, increasing acetylation, activating demethylases, and deactivating methyltransferases, providing a basis for explaining how oxygen could influence hPSC by providing a more flexible transcriptional state. Moreover, this euchromatic state at 5% oxygen coincides with a wave of transcriptional activity and a time-dependent increase in the flux of glucose-derived carbon through multiple metabolic pathways. The requirement for pluripotent cells to maintain specific metabolic, epigenetic, and transcriptional profiles implicates oxygen as a key developmental regulator through its ability to influence metabolism. These findings have broad implications because the role of oxygen in metabolism, transcription, and epigenetics has obvious implications for all aspects of pluripotent cell culture, including differentiation assays, and disease modelling.

## 4. Materials and Methods

### 4.1. Cell Culture

hPSC MEL1 and MEL2 (Australian Stem Cell Centre) were cultured in mTeSR1™ medium (Stem Cell Technologies) on PSC qualified Matrigel™- (BD Biosciences) coated tissue culture plates (BD Biosciences). Cells were cultured in humidified CB 150 incubators (Binder Inc.) at 37°C with 5% CO_2_ in air (20% oxygen), or 5% CO_2_ and 5% O_2_, and balanced with N_2_. Cell passaging took place every 5 days using Dispase (Stem Cell Technologies), and the medium was refreshed every 24 h in a biosafety cabinet. To minimize exposure to 20% oxygen conditions during handling, the medium was preequilibrated under each respective oxygen condition. All cell cultures were acclimated to 5% or 20% oxygen conditions for a minimum of 2 passages before use.

### 4.2. Cellular Bioenergetics

hPSC were treated with Y-27632 (10 *μ*M; AdipoGen) for 1 h prior to dissociation with TrypLE Select (Invitrogen) and seeded as single cells at a density of 40,000 cells/well in Seahorse XF24 assay microplates (In Vitro Technologies) and cultured at 5% or 20% oxygen. For cell number determination, a parallel seeding of 40,000 cells/well of a 96-well clear bottom plate (Falcon) in mTeSR1™ + Y-27632 was performed. After 24 h, the medium was refreshed with mTeSR1™, and after a further 24 h, cells in the assay plate were prepared for analysis. Cells were rinsed with 500 *μ*L of warmed (37°C) Seahorse XF base medium (In Vitro Technologies) containing 13.7 mM glucose, 0.392 mM sodium pyruvate, and 2.94 mM L-glutamine (the levels found in mTeSR1™) at pH 7.4. A final volume of 630 *μ*L was added to the assay plate before it was placed in a 1% CO_2_ incubator for 1 h to stabilize the pH of the assay medium and the plate. A standard mitochondrial stress test assay (Seahorse Bioscience/In Vitro Technologies) was performed with an initial basal recording followed by sequential injections of oligomycin (1 *μ*M final), carbonyl cyanide-p-trifluoromethoxyphenylhydrazone (FCCP; 50 nM final) and rotenone/antimycin A (1 *μ*M final) every 24 minutes. Cells parallel seeded on clear 96-well plates were fixed in 70% ethanol and stained with DAPI. To determine the cell number, 5 representative images were taken of the cell monolayer from each of 4 separate wells and analyzed using ImageJ, and the average cell number quantitated after adjusting for the well surface area. Assay results were normalized to the cell number, and metabolic parameters were determined using the publicly available Seahorse XF Cell Mito Stress Test Report Generator (Agilent Technologies).

### 4.3. Transmission Electron Microscopy

Approximately 4 million hPSC per treatment were processed for TEM using a protocol adapted from Underwood et al. [55]. hPSC were washed with 0.1M cacodylate buffer and then fixed for 30 min in cacodylate buffer containing 1% glutaraldehyde and 1% PFA at pH 7.4. Fixed cells were scraped and then centrifuged at 300g for 3 min and rinsed in cacodylate buffer 3 times. Pelleted cells were stained with 1% osmium tetroxide for 30 min, rinsed with cacodylate buffer, and then dehydrated through an ethanol gradient as follows: 50% ethanol for 10 min, 70% ethanol for 10 min, 90% ethanol for 10 min, 95% ethanol for 10 min, 100% ethanol for 15 min, and 100% electron microscopy grade ethanol for 30 min. Samples were then infiltrated with 200 *μ*L resin (50% absolute acetone: 50% resin) in an open tube under agitation for 2 h. Resin was refreshed and infiltrated for 4 h under vacuum, then set in 100% resin at 80°C. Samples were sliced with a microtome, fixed to TEM grids, and imaged using a Jeol 2100F transmission electron microscope with an Oxford X-MaxN 80 T EDXS detector (Oxford Instruments), an EELS Spectrometer imaging filter (Gattan), and an Orius SC1000 CCD camera (Gattan). The microscope was operated at an accelerating voltage of 80 kV.

### 4.4. In Vitro Teratoma Assay

Methylcellulose (Sigma) was resuspended to make a 10% solution in knockout serum replacement (KOSR) medium consisting of DMEM/F12+L-glutamine (Thermo Fisher) with 20% (*v*/*v*) KOSR (Thermo Fisher), 0.1 mM NEAA (Thermo Fisher), and 10,000 U PenStrep (Thermo Fisher). hPSC cultured at 20% oxygen for over 5 passages were treated with Y-27632 (10 *μ*M) for 1 h prior to dissociation with TrypLE Select. A 3 mm coat of methylcellulose was added to wells of a 24-well ultralow attachment plate (Corning) and incubated at 37°C for approximately 40 min until solidified. hPSC were harvested with 5 mL KOSR+20% FBS, then 6.25 million cells were centrifuged at 300 g and resuspended in 5 mL of KOSR+20% FBS+0.25 mL of undiluted Matrigel (Corning)+10 *μ*M Y-27632. Approximately 5,000,000 cells (400 *μ*L) were loaded into each well on top of the methylcellulose layer and the plates were centrifuged at 300g for 5 min until tight spheres of cells had formed; then, 200 *μ*L of KOSR medium+10 *μ*M Y-27632 was added and incubated overnight at 20% oxygen. After 24 h, following attachment of the spheres to the methylcellulose layer, the medium was aspirated and replaced with 400 *μ*L/well of KOSR medium+20% FBS. After a further 24 h, the medium was completely aspirated and a second 3 mm layer of methylcellulose was added on top of the cell aggregate and placed in the incubator for ~40 min. Once solidified, 400 *μ*L of KOSR medium+20% FBS was added on top of the methylcellulose layers. The medium was replaced every 48 h for 8 weeks. After 8 weeks of differentiation, *in vitro* teratomas were extracted, fixed, and stained with H&E for morphological analysis.

### 4.5. Fluxomics Using ^13^C-glucose

To determine metabolite kinetics, uniformly labeled ^13^C_6_ D-glucose (Sigma) was supplemented into glucose-free mTeSR1™ medium. Long-term 5% and 20% oxygen-adapted hPSC were treated with Y-27632 (10 *μ*M) for 1 h, dissociated with TrypLE Select, and 4 × 10^6^ cells were seeded as single cells in 100 mm dishes (Becton Dickinson) in glucose-free mTeSR1™ + ^13^C_6_ D-glucose+Y-27632 and cultured at 5% or 20% oxygen. Upon reaching confluency, the medium was changed 4 h prior to labeling, with in-house glucose-free mTeSR1™ supplemented with D-glucose. After 4 h, custom mTeSR1™+^13^C_6_ D-glucose was added to timepoint dishes 1, 2, and 4 h, which were returned to their respective incubators for 1, 2, or 4 h, respectively. Timepoint 0 h was harvested immediately. For each collection, 10 mL of spent ^13^C_6_ D-glucose medium was collected and stored at -80°C. Ice cold PBS was added to the cells prior to scraping, cell suspensions were centrifuged at 300g for 3 min, the supernatant was aspirated, and the pellets were snap frozen in liquid nitrogen before being stored at -80°C. A 1 cm^2^ area of cells was left on each plate for cell number determination.

Sample preparation and NMR spectra acquisition were performed as previously described [29]. In detail, frozen cell pellets were lysed by cytolysis using molecular grade water on ice for 20 min. Cell lysates were separated into aqueous and organic phases using a 2 : 1 chloroform : methanol solution, followed by 1-part methanol and 1-part molecular grade water with vigorous vortexing between each step, and centrifuged at 3000 g at 4°C giving rise to a biphasic solution. The aqueous and organic phases were recovered and, together with the spent medium, were concentrated by lyophilization. The aqueous and media concentrates were reconstituted in deuterated water (D_2_O; Cambridge Isotopes) with 1 mM DSS (Sigma). The organic concentrate was reconstituted in deuterated chloroform (CDCl_3_; Cambridge Isotopes) and analyzed immediately. Samples were loaded into 3 mm glass tubes (Wilmad-LabGlass) and analyzed on a 63 mm bore 800 MHz spectrometer (Varian/Agilent Technologies) using VnmrJ software (Agilent Technologies) by collecting gradient-enhanced 1D-^1^H and 2D-^1^H,^13^C-heteronuclear single-quantum coherence (cCHSQC) spectra. Acquisition and recycling time for aqueous, organic, and media samples were 6 h and 1.5 h, 4.5 h and 1.25 h, and 0.5 h and 0.125 h, respectively. Spectra were processed using Mnova (Mestrelab Research), by integrating peaks relative to DSS or CDCl_3_. Chemical shift data from the Human Metabolome Database and Biological Magnetic Resonance Data Bank [56, 57] were used to identify metabolites, and integrals were transformed to mass quantities using standard curves. Mass quantities for each metabolite were standardized to 25 million cells and plotted as flux in nmol over time (h).

### 4.6. Immunofluorescence

For the quantification of histone acetylation and methylation markers, hPSC were fixed in PFA for 15 min, permeabilized using 1 M HCl for 20 min, and blocked with 0.1% Triton X-100 and 5% species-specific serum in PBS for 1 h. Primary antibodies were incubated at 4°C for 24 h at the following concentrations: H3K9ac (Abcam) at 1 : 500, H3K27ac (Abcam) at 1 : 1000, and H3K27me3 (Abcam) at 1 : 250. Secondary antibodies Alexa Fluor 488 or 568 (Invitrogen) were used at 1 : 1000 and incubated at RT for 45 min. Nuclei were counterstained with 300 nM DAPI for 5 min at RT. For each marker of interest, exposure times for DAPI and primary were kept constant. Five representative colonies were imaged per treatment, and 10 cells were randomly selected per colony for fluorescence intensity analysis using ImageJ. Corrected total cell fluorescence (CTCF) was calculated using the following formula [58]: CTCF = integrated density − (area of nucleus × mean fluorescence of background readings). CTCF was then further normalized to the DAPI intensity for each selected nucleus.

### 4.7. Flow Cytometry

To determine mitochondrial ROS production, hPSC were dissociated using TrypLE Select, centrifuged at 300g, incubated in mTeSR1™ with the MitoSOX Red superoxide indicator (1 *μ*M, Invitrogen) for 20 min at 37°C, counterstained with 300 nM DAPI, and washed twice with PBS. Cells were then run on a Beckman Coulter CyAn analyzer (Beckman Coulter), where a minimum of ten thousand live cell events were acquired using DAPI gating for each sample. Flow density plots were generated using Summit 4.3 software (Beckman Coulter). Mean log_10_ fluorescence data values were recorded and normalized for the statistical analysis.

### 4.8. qRT-PCR

Total RNA was isolated from hPSC using TRIzol (Invitrogen). RNA was isolated using a chloroform-induced triphasic solution before DNase treatment using DNase-I (Ambion). cDNA was synthesized from 1 *μ*g of RNA using M-MLV Reverse Transcriptase (Promega). Triplicate reactions of each sample were run using EvaGreen Master Mix (Solis BioDyne) on a ViiA7™ thermocycler (Invitrogen). No-template control samples with nuclease-free water in place of cDNA were included for every primer set, and a –RT control was included to confirm appropriate cDNA synthesis. The relative abundance of each gene was normalized to that of *RPLP0* and analyzed using Q-Gene which is based on the ΔΔCt method. Standard curves were generated for each gene to control for primer-specific efficiencies. All PCR products were verified by sequencing. See Supplementary Table 2 for primers.

### 4.9. RNA-seq

RNA from MEL1 and MEL2 hPSC cultured at 5% and 20% oxygen was isolated using the RNeasy Plus Mini Kit (Qiagen). The RNA integrity numbers (RIN) of 10 for all samples were determined using the Agilent 2100 Bioanalyzer (Agilent Technologies). RNA was analyzed on the Illumina HiSeq 2500 Sequencing System (Illumina) at the Australian Genome Research Facility (AGRF) using 1 *μ*g of total RNA as input. Sequencing was performed with 12 samples per lane, using 50 bp single-end reads. The quality of resultant raw FASTQ data files was assessed with FastQC. Data files were uploaded to the Galaxy platform [59] at https://usegalaxy.org/ and processed using the following pipeline: FASTQ Groomer to convert raw Illumina FASTQ files to Sanger Quality score format, Trim Galore! to trim the 13 bp of Illumina standard adapters, RNA STAR to align reads to the UCSC GRCh37/hg19 human reference genome, featureCounts to measure gene expression, and DESeq2 to identify DEGs. GO analysis of Biological Function terms and KEGG pathways were determined using DAVID. DEGs were submitted to NetworkAnalyst [38] to visualize zero-order protein-protein interaction (PPI) networks using the STRING interactome with a confidence score cut-off of 900.

### 4.10. Statistical Analyses

All data are presented as the mean ± SEM (*p* < 0.05). Statistical analyses were performed using Student's *t*-test to calculate differences between two groups, or following a two-factor ANOVA, significant main effects and interactions were further analyzed using simple main effects calculated using the MS residual from the initial ANOVA. All analyses were performed in Prism 7 (GraphPad) and unless otherwise stated *n* = 3, where *n* is the number of biological replicates.

## Data Availability

The Gene Expression Omnibus accession number for the RNA-seq dataset reported in this paper is GSE117966.

## Supplementary Materials

Supplementary 1 Supplementary Figure 1: carbon tracing through metabolic pathways. Metabolite pathways organized into functional groups contributing to the production of glycogen, glutathione, pyruvate, alanine, acetate, and lactate. Figures show the absolute levels of intracellular glucose derivatives in MEL2 hPSC at 0.5, 1, 2, and 4 hours when cultured under 5% and 20% oxygen. Lactate is plotted on the right *y*-axes. Supplementary Figure 2: RNA-seq volcano plots of the hPSC response to oxygen. Plots from left to right and top to bottom are the MEL1 hPSC transcriptional response to 5% and 20% oxygen and the transcriptional differences between hPSC lines at 5% oxygen, at 20% oxygen, and when the oxygen treatments are pooled. Red genes indicate a fold change value greater than 2 and an adjusted *p* value (Benjamini FDR) less than 0.05. Supplementary Figure 3: hPSC transcriptional response to 5% and 20% oxygen. (A) Pluripotency markers are generally not impacted by oxygen. (B) HIF-1 target genes show a strong transcriptional activation at 5% oxygen. Only statistics with a Benjamini score of <0.05 (p-adj) are shown. All assays performed in biological triplicate. ^∗∗∗∗^ *p* < 0.0001, ^∗∗∗^ *p* < 0.001, ^∗∗^ *p* < 0.01, and ^∗^ *p* < 0.05 for Benjamini scores. Supplementary Figure 4: Enriched GO and KEGG pathways due to oxygen. MEL1 and MEL2 hPSC GO and KEGG pathways upregulated after 5% and 20% oxygen culture. The contributing numbers of gene hits for each pathway are given with the ends of each bar. Only terms with a Benjamini score of <0.05 (p-adj) are shown. All assays performed in biological triplicate. ^∗∗∗∗^ *p* < 0.0001, ^∗∗∗^ *p* < 0.001, ^∗∗^ *p* < 0.01, and ^∗^ *p* < 0.05 for Benjamini scores. Supplementary Figure 5: RNA-seq minimum-order networks comparing hPSC lines. (A) Differentially expressed genes in hPSC lines (MEL1, MEL2) cultured at 5% oxygen were connected based on known protein:protein interactions (https://www.innatedb.ca/). Red nodes are upregulated in MEL1 hPSC; green nodes are upregulated in MEL2 hPSC. Grey nodes have known interactions with the seeds but were not regulated. Green/red intensity indicates the degree of fold change as indicated in the figure. (B) 5% oxygen cultured KEGG pathways upregulated in MEL1 and MEL2 hPSC based on the minimum-order network in Supplementary Figure 2A. (C) 20% oxygen minimum-order, protein:protein interaction network. (D) 20% oxygen KEGG pathways upregulated in MEL1 and MEL2 hPSC based on the minimum-order network in Supplementary Figure 2C. Supplementary Figure 6: methyltransferases and lysine demethylases synergistically reduce methylation at 5% oxygen. (A) Extracellular and intracellular levels of glycolytic metabolites and glycolytic enzymes in hPSC. (B) Intracellular levels of metabolites and enzymes in and related to the serine/glycine biosynthesis pathway. (C) Intercellular metabolite levels of transsulphuration pathway metabolites. (D) Expression of metabolites and transcripts for enzymes, in the folate cycle. (E) Expression of metabolites and transcripts for enzymes, in the methionine cycle. (F) Level of H3K27 trimethylation and transcripts for lysine demethylases and methyltransferases. All contributing assays performed in a minimum of biological triplicate. Bolded text indicates a significant increase (green) or decrease (red) in hPSC metabolite/transcript/methylation level at 5% relative to 20% oxygen culture. Bolded black text indicates a nonsignificant result for an assessed parameter. Unbolded text indicates a parameter that was not assessed. Supplementary Table 2: related to experimental procedures. Human PCR primers for the serine/glycine biosynthesis pathway.

Supplementary 2 Supplementary Table 1: an attached excel file containing qPCR data relating to Supplementary Figure 5 and the 4 comparisons performed on RNA-seq data comparing MEL1 hPSC at 5% and 20% oxygen, MEL2 hPSC at 5% and 20% oxygen, MEL1 and MEL2 hPSC at 5% oxygen, and MEL1 and MEL2 hPSC at 20% oxygen.
