# Supplementary material for: Oxygen Regulates Human Pluripotent Stem Cell Metabolic Flux
Source: Stem Cells Int. 2019 May 19;2019:8195614. doi: 10.1155/2019/8195614 (PMC6545818; doi:10.1155/2019/8195614)
Supplement: Supplementary 1 — Supplementary Figure 1: carbon tracing through metabolic pathways. Metabolite pathways organized into functional groups contributing to the production of glycogen, glutathione, pyruvate, alanine, acetate, and lactate. Figures show the absolute levels of intracellular glucose derivatives in MEL2 hPSC at 0.5, 1, 2, and 4 hours when cultured under 5% and 20% oxygen. Lactate is plotted on the right y-axes. Supplementary Figure 2: RNA-seq volcano plots of the hPSC response to oxygen. Plots from left to right and top to bottom are the MEL1 hPSC transcriptional response to 5% and 20% oxygen and the transcriptional differences between hPSC lines at 5% oxygen, at 20% oxygen, and when the oxygen treatments are pooled. Red genes indicate a fold change value greater than 2 and an adjusted p value (Benjamini FDR) less than 0.05. Supplementary Figure 3: hPSC transcriptional response to 5% and 20% oxygen. (A) Pluripotency markers are generally not impacted by oxygen. (B) HIF-1 target genes show a strong transcriptional activation at 5% oxygen. Only statistics with a Benjamini score of <0.05 (p-adj) are shown. All assays performed in biological triplicate. ∗∗∗∗ p < 0.0001, ∗∗∗ p < 0.001, ∗∗ p < 0.01, and ∗ p < 0.05 for Benjamini scores. Supplementary Figure 4: Enriched GO and KEGG pathways due to oxygen. MEL1 and MEL2 hPSC GO and KEGG pathways upregulated after 5% and 20% oxygen culture. The contributing numbers of gene hits for each pathway are given with the ends of each bar. Only terms with a Benjamini score of <0.05 (p-adj) are shown. All assays performed in biological triplicate. ∗∗∗∗ p < 0.0001, ∗∗∗ p < 0.001, ∗∗ p < 0.01, and ∗ p < 0.05 for Benjamini scores. Supplementary Figure 5: RNA-seq minimum-order networks comparing hPSC lines. (A) Differentially expressed genes in hPSC lines (MEL1, MEL2) cultured at 5% oxygen were connected based on known protein:protein interactions (https://www.innatedb.ca/). Red nodes are upregulated in MEL1 hPSC; green nodes are upregulated in MEL [file 8195614.f1.pdf]

Supplementary Figures and Tables

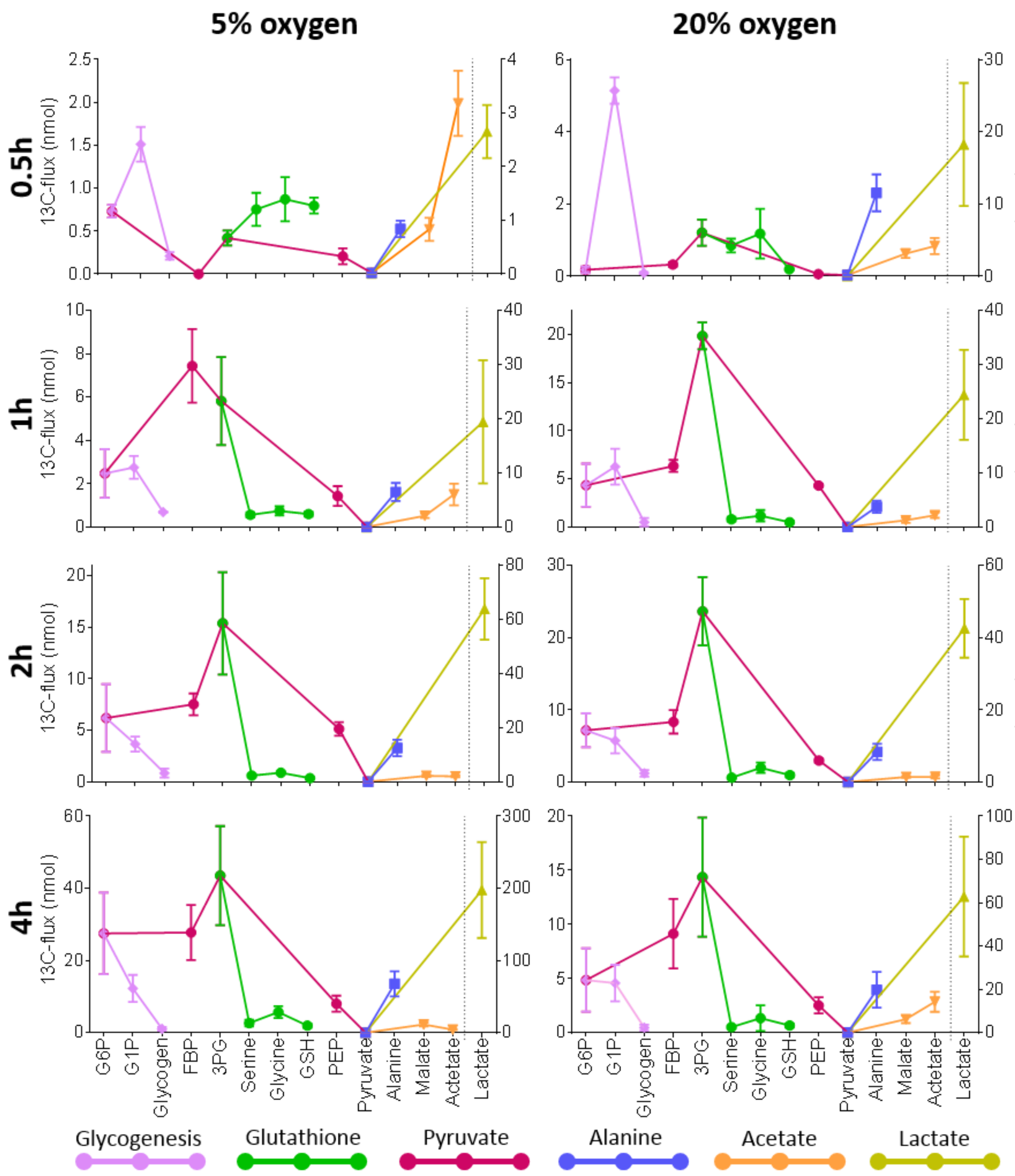

Supplementary Figure 1. Carbon tracing through metabolic pathways. Metabolite pathways organised into functional groups contributing to the production of: glycogen, glutathione, pyruvate, alanine, acetate, and lactate. Figures show the absolute levels of intracellular glucose derivatives in MEL2 hPSC at 0.5, 1, 2 and 4 hours when cultured under 5% and 20% oxygen. Lactate is plotted on the right y-axes.

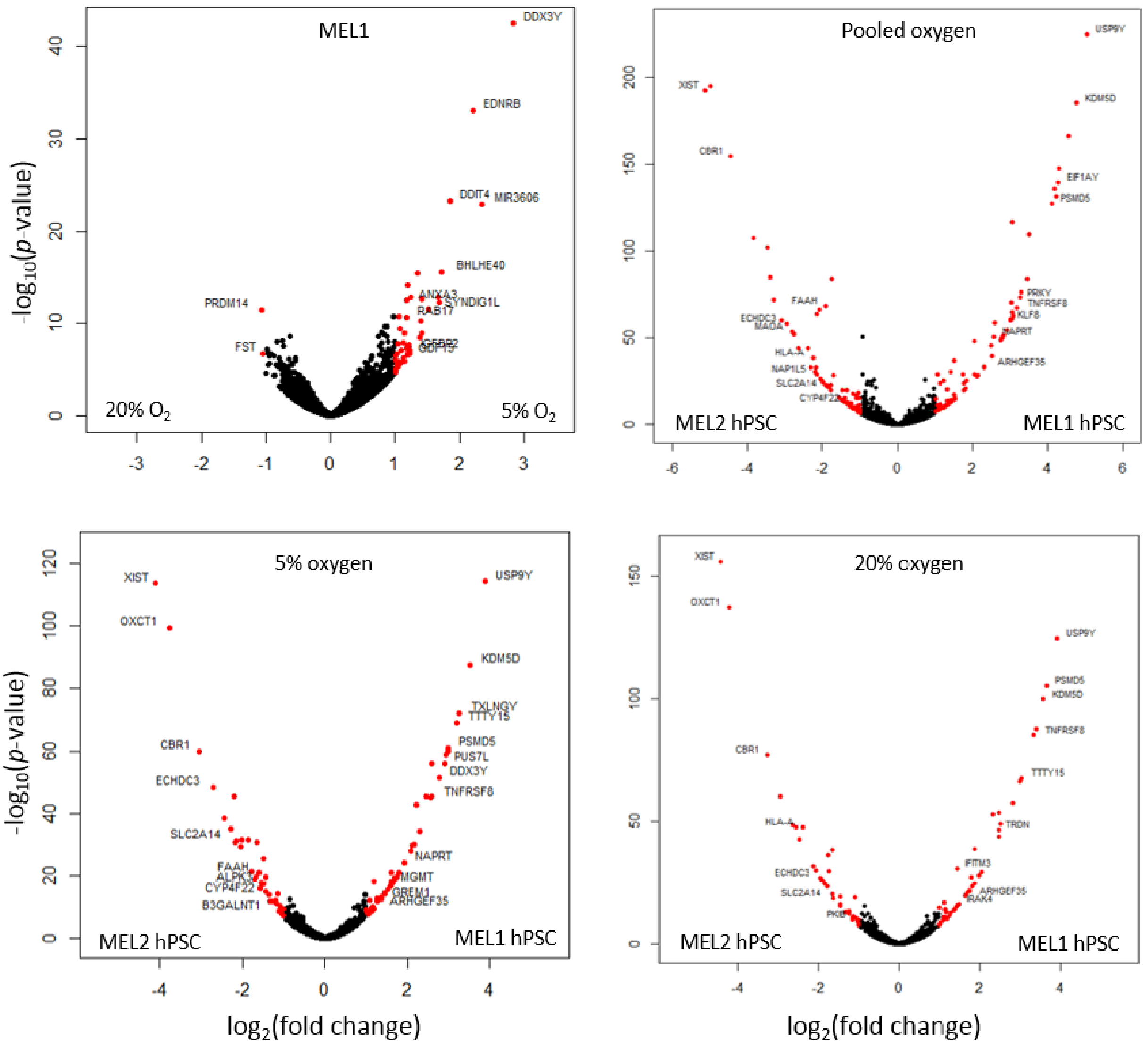

Supplementary Figure 2. RNA-seq volcano plots of the hPSC response to oxygen. Plots from left to right, top to bottom are: the MEL1 hPSC transcriptional response to 5% and 205 oxygen, the transcriptional differences between hPSC lines at 5% oxygen, at 20% oxygen, and when the oxygen treatments are pooled. Red genes indicate a fold change value greater than 2 and an adjusted p-value (Benjaminni FDR) less than 0.05.

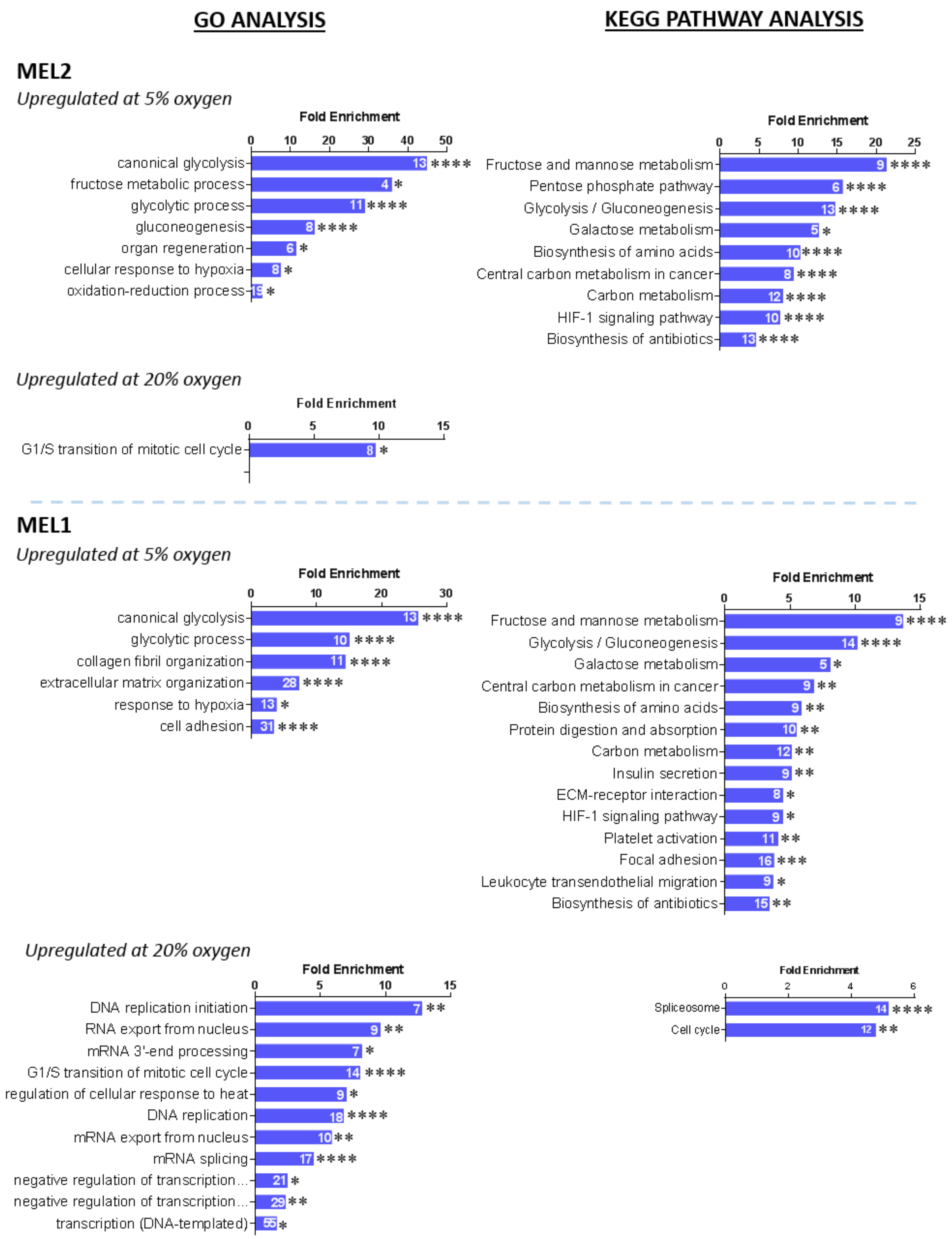
GO ANALYSIS
KEGG PATHWAY ANALYSIS
MEL2
Upregulated at 5% oxygen
Fold Enrichment
0 10 20 30 40 50
canonical glycolysis 13 ****
fructose metabolic process 4 *
glycolytic process 11 ****
gluconeogenesis 8 ****
organ regeneration 6 *
cellular response to hypoxia 8 *
oxidation-reduction process 19 *
Fold Enrichment
0 5 10 15 20 25
Fructose and mannose metabolism 9 ****
Pentose phosphate pathway 6 ****
Glycolysis / Gluconeogenesis 13 ****
Galactose metabolism 5 *
Biosynthesis of amino acids 10 ****
Central carbon metabolism in cancer 8 ****
Carbon metabolism 12 ****
HIF-1 signaling pathway 10 ****
Biosynthesis of antibiotics 13 ****
Upregulated at 20% oxygen
Fold Enrichment
0 5 10 15
G1/S transition of mitotic cell cycle 8 *
MEL1
Upregulated at 5% oxygen
Fold Enrichment
0 10 20 30
canonical glycolysis 13 ****
glycolytic process 10 ****
collagen fibril organization 11 ****
extracellular matrix organization 28 ****
response to hypoxia 13 *
cell adhesion 31 ****
Fold Enrichment
0 5 10 15
Fructose and mannose metabolism 9 ****
Glycolysis / Gluconeogenesis 14 ****
Galactose metabolism 5 *
Central carbon metabolism in cancer 9 **
Biosynthesis of amino acids 9 **
Protein digestion and absorption 10 **
Carbon metabolism 12 **
Insulin secretion 9 **
ECM-receptor interaction 8 *
HIF-1 signaling pathway 9 *
Platelet activation 11 **
Focal adhesion 16 ***
Leukocyte transendothelial migration 9 *
Biosynthesis of antibiotics 15 **
Upregulated at 20% oxygen
Fold Enrichment
0 5 10 15
DNA replication initiation 7 **
RNA export from nucleus 9 **
mRNA 3'-end processing 7 *
G1/S transition of mitotic cell cycle 14 ****
regulation of cellular response to heat 9 *
DNA replication 18 ****
mRNA export from nucleus 10 **
mRNA splicing 17 ****
negative regulation of transcription... 21 *
negative regulation of transcription... 29 **
transcription (DNA-templated) 55 *
Fold Enrichment
0 2 4 6
Spliceosome 14 ****
Cell cycle 12 **

Supplementary Figure 3. Enriched GO and KEGG pathways due to oxygen. MEL1 and MEL2 hPSC GO and KEGG pathways upregulated after 5% and 20% oxygen culture. The contributing number of gene hits for each pathway are given with the ends of each bar. Only terms with a Benjamini score of < 0.05 (*p*-adj) are shown. All assays performed in biological triplicate. ****$p < 0.0001$, ***$p < 0.001$, **$p < 0.01$, *$p < 0.05$ for Benjamini score.

A

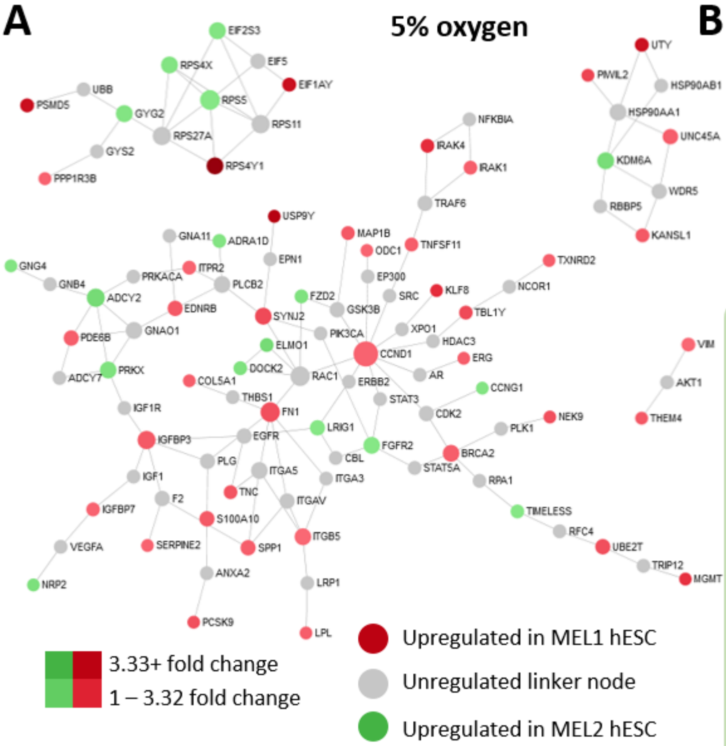

B

Upregulated in MEL1 hESC

Pathway	Hits	P.Value
ECM-receptor interaction	5	3.42E-05
Focal adhesion	6	0.000237
Toll-like receptor signaling pathway	3	0.00999
Measles	3	0.0115
Fanconi anemia pathway	2	0.0141
Alzheimer's disease	2	0.0217
Leishmaniasis	2	0.0234
Pertussis	2	0.0243
p53 signaling pathway	2	0.0399
Pancreatic cancer	2	0.0409
Phosphatidylinositol signaling system	2	0.0476

Upregulated in MEL2 hESC

Pathway	Hits	P.Value
Chemokine signaling pathway	5	2.18E-05
Salivary secretion	3	0.000123
Morphine addiction	3	0.000174
Retrograde endocannabinoid signaling	3	0.000226
GABAergic synapse	3	0.000313
Cholinergic synapse	3	0.000877
Glutamatergic synapse	3	0.00105
Melanogenesis	3	0.00105
Vascular smooth muscle contraction	3	0.00131
Bile secretion	2	0.00162
Calcium signaling pathway	3	0.00524
Gastric acid secretion	2	0.00595
Dilated cardiomyopathy	2	0.0113
Progesterone-mediated oocyte maturation	2	0.0119
Serotonergic synapse	2	0.0143
Gap junction	2	0.0146
GnRH signaling pathway	2	0.0162
Oocyte meiosis	2	0.021
Dopaminergic synapse	2	0.0273
Wnt signaling pathway	2	0.036

C

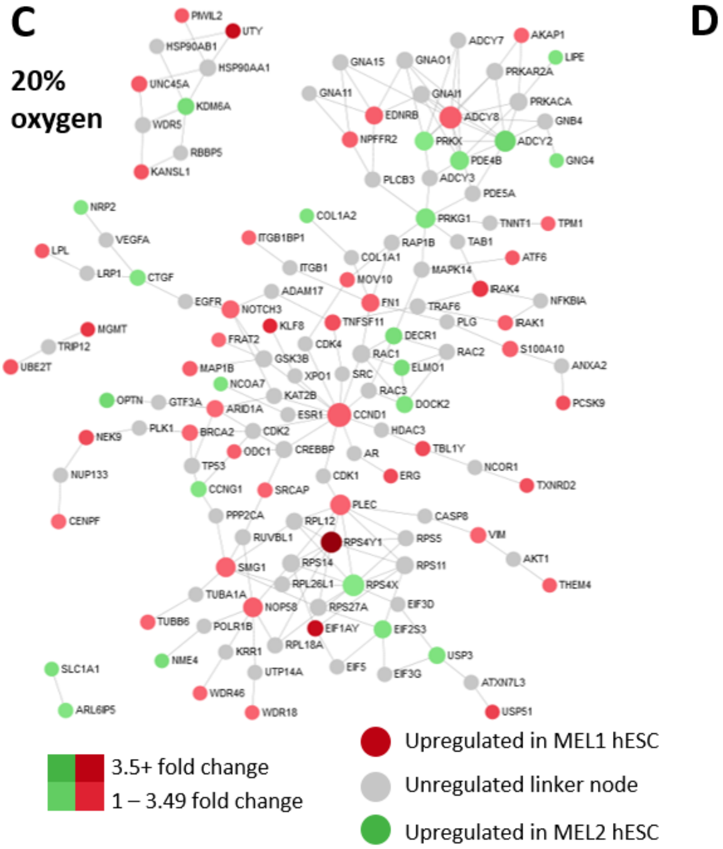

D

Upregulated in MEL1 hESC

Pathway	Hits	P.Value
Measles	3	0.0101
Fanconi anemia pathway	2	0.0129
Alzheimer's disease	2	0.0199
Leishmaniasis	2	0.0214
Pertussis	2	0.0222
Wnt signaling pathway	3	0.0253
Pancreatic cancer	2	0.0376
Dilated cardiomyopathy	2	0.0469
Small cell lung cancer	2	0.0491

Upregulated in MEL2 hESC

Pathway	Hits	P.Value
Chemokine signaling pathway	5	6.40E-05
Salivary secretion	3	0.000222
Morphine addiction	3	0.000314
Retrograde endocannabinoid signaling	3	0.000406
GABAergic synapse	3	0.000563
Gap junction	3	0.00129
Cholinergic synapse	3	0.00156
Glutamatergic synapse	3	0.00186
Vascular smooth muscle contraction	3	0.00232
Bile secretion	2	0.00235
Purine metabolism	3	0.00724
Gastric acid secretion	2	0.0086
Dilated cardiomyopathy	2	0.0162
Progesterone-mediated oocyte maturation	2	0.017
Serotonergic synapse	2	0.0204
GnRH signaling pathway	2	0.0231
Melanogenesis	2	0.0264
Oocyte meiosis	2	0.0299
Dopaminergic synapse	2	0.0386
Insulin signaling pathway	2	0.0463

Supplementary Figure 4. RNA-Seq minimum-order networks comparing hPSC lines. (A) Differentially expressed genes in hPSC lines (MEL1, MEL2) cultured at 5%oxygen were connected based on known protein:protein interactions (www.innatebd.ca). Red nodes are upregulated in MEL1 hPSC; green nodes are upregulated in MEL2 hPSC. Grey nodes have known interactions with the seeds but were not regulated. Green/red intensity indicates the degree of fold change as indicated in the figure. (B) 5% oxygen cultured KEGG pathways upregulated in MEL1 and MEL2 hPSC based on the minimum-order network in Supplementary Fig. 2A. (C) 20% oxygen minimum-order, protein:protein interaction network. (D) 20% oxygen KEGG pathways upregulated in MEL1 and MEL2 hPSC based on the minimum-order network in Supplementary Fig. 2C.

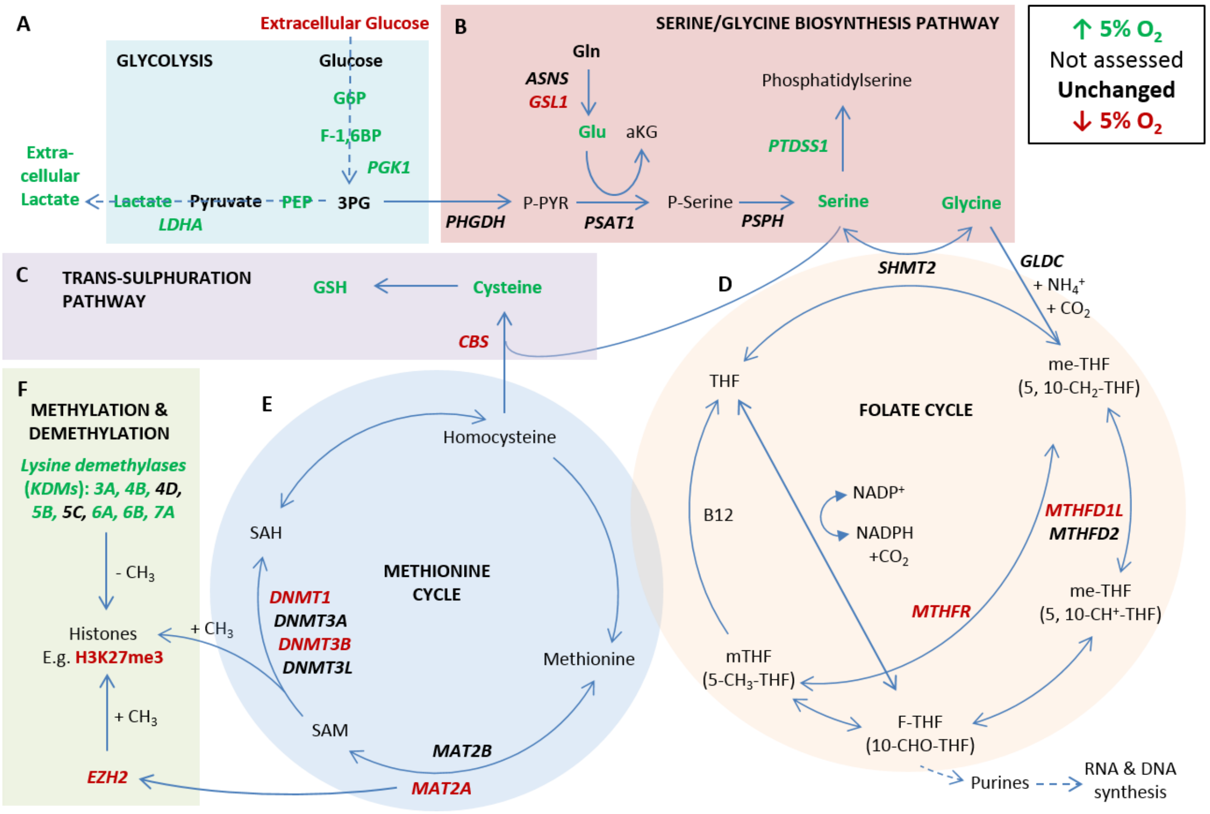

Supplementary Figure 5. Methyltransferases and lysine demethylases synergistically reduce methylation at 5% oxygen. (A) Extracellular and intracellular levels of glycolytic metabolites and glycolytic enzymes in hPSC. (B) Intracellular levels of metabolites and enzymes in and related to the serine/glycine biosynthesis pathway. (C) Intercellular metabolite levels of trans-sulphuration pathway metabolites. (D) Expression of metabolites, and transcripts for enzymes, in the folate cycle. (E) Expression of metabolites, and transcripts for enzymes, in the methionine cycle. (F) Level of H3K27 trimethylation, and transcripts for lysine demethylases and methyltransferases. All contributing assays performed in a minimum of biological triplicate. Bolded text indicates a significant increase (green) or decrease (red) in hPSC metabolite/transcript/methylation level at 5% relative to 20% oxygen culture. Bolded black text indicates a non-significant result for an assessed parameter. Unbolded text indicates a parameter that was not assessed.

Supplementary Tables

Supplementary Table 1 is an attached excel file containing qPCR data relating to Supplementary Figure 5, and the 4 comparisons performed on RNA-Seq data comparing MEL1 hPSC at 5% and 20% oxygen, MEL2 hPSC at 5% and 20% oxygen, MEL1 and MEL2 hPSC at 5% oxygen, and MEL1 and MEL2 hPSC at 20% oxygen.

Supplementary Table 2. Related to Experimental Procedures. Human PCR primers for the serine/glycine biosynthesis pathway.

Gene	Size	Forward	Reverse
RPLP0	106	GGAGGGTGTCCGCAATGTT	CAAGGCCAGGACTCGTTTGT
ASNS	64	GCAGCTGAAAGAAGCCCAAGT	TGTCTTCCATGCCAATTGCA
CBS	151	TCATCGTGATGCCAGAGAAG	TTGGGGATTTCGTTCTTCAG
GLDC	173	AGGGAGCAACACATTCGGAG	GCTCGCTTGAGACCTTCTGA
GLS1	114	AGGGTCTGTTACCTAGCTTGG	ACGTTCGCAATCCTGTAGATTT
MAT2A	287	CCACGAGGCGTTCATCGAGG	AAGTCTTGTAGTCAAAACCT
MAT2B	121	TGGGGAGCACTTGAAAGAG	CTTAGCGGCAACATGGG
MTHFD1L	164	GGGACTCCATCGTCAGAGAA	GTTCAGACCAGCCTCCTCAG
MTFFD2	189	GCCTCCTTGTTCAGTTGCCT	TTGGAATGCCAGTTCGCTTG
MTHFR	173	CGGGGAGACCCAATAGGTGA	AAGTGCTTCAGGTCAGCCTC
PSAT1	218	AGAATCTTGTGCGGGAATTG	CCCAAGTTTAGGGTGAACGA
PHGDH	107	ATCTCTCACGGGGGTTGTG	AGGCTCGCATCAGTGTCC
PTDSS1	220	ATGTGATCACCTGGGAGAGG	CCATTGCACAACAGGATGTC
SHMT2	51	AGTCTATGCCCTATAAGCTCAACC	GCCGGAAAAGTCGAGCAGT
